# Monthly Summary

**Published:** 1899-05

**Authors:** 


### Monthly Summary.

BROKE A TOOTH AND BLED TO DEATH.—William Oswald Billups, the thirteen year old grandson of Major W. J. Houston, of Decatur, died yesterday morning. His death was the result of breaking one of his upper jaw teeth while eating dinner, Thursday, and from the trivial hurt he bled to death.

The case has few parallels in the annals of medicine, albeit enough instances of the sort have been recorded by physicians to establish beyond all doubt that deaths have been similarly caused before.

Billups belonged to a class of physical idiocrats popularly known as "bleeders"—a person, as the word implies, affected with a predisposition to bleed. That blood lacks the peculiar property that causes coagulation. Hence, the slightest wounds are dangerous to their lives, for once a flow of blood is started, to check it is almost impossible.

That the boy belonged to this class; his mother, Mrs. Robert R. Billups, and his grandfather, Major Houston, were aware. Some years ago a brother of his sustained a slight cut on the index finger, and the only way in which the resulting loss of the vital fluid could be stopped was by the use of a turniquet. They therefore supposed that the other son was similarly affected.

When a few weeks ago he began shedding his milk teeth, and developing the permanent set. he was cautioned not to pull any of the old ones out, no matter how loose they became —the idea being to let the new comers gradually and bloodlessly usurp the places of the old. He obeyed this injunction rigidly, and all went well until Thursday. On that day, however, while eating dinner he broke out one of the loosened old teeth. A great flow of blood instantly ensued, and never ceased until death claimed him.

At first various home remedies were resorted to to stop the bleeding. These all failing, physicians were summoned—Drs. Goss and Ramsey, of Decatur, and Dr. Elkin, of Atlanta. Their efforts, too, were fruitless.

Last Thursday aftnrnoon the boy's entire gum dropped out. This horrible complication increased the effected surface many times, and therefore enormously increased the flow of blood. Death began to approach rapidly, then, and yesterday morning at one o'clock the victim breathed his last.

The boy's life-blood did not, as in ordinary cases, spurt out in a jet or stream. Instead, It oozed out slowly, but with unconquerable steadiness. And so completely drained was his system, that after death his body was as white as Italian marble.

Young Billups was the son of the late R. R. Billups, who died about three years ago, and who was for a number of years before his demise, in the railway service, and well known not only in Atlanta, but throughout this section of the State. His mother is the daughter of Major W. R. Houston, of Decatur, one of DeKalb's most prominent citizens.—Atlanta Constitution, January 21st, 1899.

FATALITIES FROM ANESTHETICS.—It is with great difficulty that figures relative to mortalities from anesthetics are obtained, because neither the local nor general government makes any effort to especially chronicle the deaths from anesthetics, and in many instances death is attributed to some other cause. Dr. Ernest Hankel, in his recent book, "Handbuch Der Inhalation Anesthetics," states that he has made a very careful investigation into the merits of the several sleep producing agents, and his research yields the following statistics :

Pental in 213 cases, one death.
Chloroform in 2,039 cases, one death.
Ether in 5,090 cases, one death.
Ethyl Bromyd in 5,228 cases, one death.
Nitrous oxid in 500,000 cases, one death.

—*Pacific Medico Dental Gazette.*

---

PYORRHŒA ALVEOLARIS TREATMENT.—My treatment in all cases of pyorrhœa is the same, and with it I have never failed in effecting a speedy and permanent cure in the most difficult cases. I begin by extracting all the tartar from the necks and fangs of the teeth; wash out thoroughly with warm water all around the teeth. Give the patient a mouth wash, such as a 50 per cent. solution of listerine, or 50 per cent. euthymol. I then prescribe Lithia tablets to be taken three times daily in a tumbler full of water; with instructions to patient not to eat rich food, especially avoid fat meats. I urge my patient to be prompt and call every other day, at which time I syringe out around the teeth thoroughly with euthymol. The lythia eliminates the uric acid from the system and renovates the kidneys, accompanied with the wash, the mouth soon heals and becomes once more in a state of health.—R. L. Ready, in *Dental Century.*

A Rubber Substitute from Corn.—We have received a sample of a rubber substitute made from corn. It is made from the oil derived from corn, and by vulcanizing it in connection with an equal quantity of crude India rubber, a substitute is produced; which, for certain purposes, is equal to the best gum rubber at a greatly lessened cost. The new corn rubber is claimed to possess all the essential qualities of Para rubber, including resiliency, and the discovery has been hailed with delight in the corn-growing states of the West. The manufacturers claim that the fact that corn oil does not oxidize readily makes this product of great value, since it is not affected by oxidation, so that product manufactured from it will always remain pliable and do not crack, as those made from other substitutes. This interesting substitute for rubber is very dark brown, or black, and it easily rubs off in light brown rolls. It is at present sold as low as six cents a pound. It is manufactured by the Glucose Sugar Refining Company, of Chicago, Ill.—*Scientific American.*

---

Sensitive Dentine.—Dr. C. B. Rohland, of Illinois, says: "By adding just sufficient carbolic crystals to cocain hydrochlorate, and rubbing together with a spatula until the cocain is dissolved, a thick syrup is obtained, which is escharotic, antiseptic, obtundent. With this he often obtains most gratifying results in the treatment of sensitive dentine in cavities of decay. It should be used with the rubber-dam, dryness to the verge of disiccation secured, applied warm, and treated in situ with the hot.air syringe, so hot as can be borne, and again dried before excavating. If one application fails to give the desired result, two almost invariably will be effective."—*Dental Brief.*

---

Eucaine.—Eucaine provokes hyperemia at the point of application, and should be used with caution when hemorrhage is to be feared. Five minims of a ten per cent. solution are sufficient to inject into the gum for the painless extraction of eeth.—Wolff, *Dental Digest.*

REPAIRING A GOLD CROWN.—Just suppose you have made a gold crown, and in finishing you go through the shell making an unsightly hole. If you undertake to solder this the chances are that you will have three or four holes caused by the solder melting out at the joints. To prevent this trouble, paint the crown all over the outside with whiting mixed thin except around the hole which you wish to repair, fill this with a plug made from gold foil, touch it up with a drop of borax water, and put a bit of gold solder inside, heat it with blow-pipe and success will be the result.—E. A. Randall, *Dominion Dental Journal.*

---

SMOKERS' TEETH.—It has been found that the teeth of smokers are less liable to decay than those of non-smokers, and it has been found by scientific research that leptothrix buccalis and the other germs found in the mouth are rendered innocuous by tobacco smoke, and it is an established fact that it also entirely destroys or retards the development of the bacillus of cholera, of anthax, and of pneumonia.—*Weekly Dentist.*

---

WHERE PYROZONE IS EFFECTIVE.—A little 25 per cent. solution of pyrozone on some cotton wound on a broach and put up the canal will instantly stop the bleeding from extirpated pulp so you could proceed to dry the canal and fill it at once. When trying to fit a crown to the root of a tooth, and the gums are bleeding and oozing so that you cannot get it dry enough to attach the crown. apply 25 per cent. pyrozone on a lock of cotton.—L. West, in *Items.*

ASEPSIS.—The ordinary scrubbing the hands with soap and water and then soaking them in a 1-1000 bichloride solution has been proven by bacteriologists as insufficient, and that to render them sterile they must be equally scrubbed with alcohol or either for a full minute to remove the oil from the pores of the skin; then the bichloride solution can have an equal action over all the surface. If the operator has operated or dressed a wound discharging pus, he should sterilize the hands further by soaking them in potassium permanganate solution, followed by an acid to neutralize the color left on the skin. When the hands are finally cleaned, in Europe they have adopted the habit of placing the elbows at the sides and holding the hands up and forward until the operation is commenced, to insure them from contact with anything.

Numerous experiments have demonstrated that the immersion of instruments for five minutes in boiling water is sufficient for their complete sterilization. If they come in contact with fetid or gangrenous substances they should be carefully cleaned, then boiled for fully five minutes before using again; carbonate of soda added to the boiling water sufficient to make a one per cent. solution prevents the instruments from rusting before again being used. I prefer gauze to sponge in operations, and I think sponges can be safely used only in large hospitals where everything is so ordered as to insure not only their proper sterilization, but equally their preservation in that condition.—Sherwood Dunn, *Review.*

---

FILLING FRAIL TEETH.—We should remember when dealing with frail teeth, having large cavities, that great masses of gold placed in contact with frail enamel walls must result in disaster from the mere effects of expansion caused by extremes of heat and cold. At one moment we drink ice water, bringing the temperature of the teeth and gold fillings down to thirty, and then we deluge the mouth with scalding hot tea, the result being a rapid and unequal rise of temperature, the gold responding and expanding to a greater degree than the enamel.—Dr. Hill, *Items.*

Suggestions on Use of the Hypodermic Syringe.—Drop a small piece of absorbent cotton into the fluid to be drawn into the syringe. Press the syringe against the cotton and the syringe will be filled with a filtered solution; no specks to stop the syringe, and less risk of after irritation at the point of puncture. Hold the syringe so that the beveled surface of the needle's point shall be firmly pressed against the skin. If the syringe is held at a right angle, the least puncture of the mere point of the needle will permit the fluid to pass under the scarf skin on a firm pressure of the piston. A white spot marks the success of the dose. We have thus caused the least pain. If slowly and carefully done no pain whatever is felt. The tissues are wounded the least and absorption is hastened. Always hold the syringe so that the needle points outward and on having introduced a mere drop wait a few seconds for a sedative effect on the tissues, then slowly push the piston home.

If the needle becomes stopped, introduce the wire at the point of the needle, as the plug can be forced more easily in thc direction from which it came.

If the needle be stopped by a vegetable substance hold the needle in the flame of the gas or the lamp and the plug will burn out.—"American Medical Compound."

---

A Low Fusible Metal.—Here is one that it is said can be, when melted, handled with the fingers and poured into a plaster mould, etc.

| | |
|---|---|
| Bismuth, - - | 48 parts. |
| Cadmium, - - | 13 " |
| Tin, - - - | 19 " |

—"British Journal of Dental Science."

---

Dental fees must often take the same course as a basis of settlement as the physician's fees. Discrimination is constant and necessary on account of the fact that all are subject to dental disease and only a small portion of the people are rich.

